# Proteostasis Deregulation in Neurodegeneration and Its Link with Stress Granules: Focus on the Scaffold and Ribosomal Protein RACK1

**DOI:** 10.3390/cells11162590

**Published:** 2022-08-19

**Authors:** Mirco Masi, Alessandro Attanzio, Marco Racchi, Benjamin Wolozin, Sofia Borella, Fabrizio Biundo, Erica Buoso

**Affiliations:** 1 Department of Drug Sciences, University of Pavia, Viale Taramelli 12/14, 27100 Pavia, Italy; 2 School for Advanced Studies IUSS, University School for Advanced Studies IUSS, Piazza della Vittoria 15, 27100 Pavia, Italy; 3 Department of Biological, Chemical and Pharmaceutical Sciences and Technologies, University of Palermo, Via Archirafi 28, 90123 Palermo, Italy; 4 Department of Pharmacology and Experimental Therapeutics, Boston University School of Medicine, Boston, MA 02118, USA; 5 Department of Developmental and Molecular Biology, Albert Einstein College of Medicine, 1300 Morris Park Ave, Bronx, NY 10461, USA

**Keywords:** neurodegeneration, RNA, proteostasis, translation, stress granules, RACK1

## Abstract

The role of protein misfolding, deposition, and clearance has been the dominant topic in the last decades of investigation in the field of neurodegeneration. The impairment of protein synthesis, along with RNA metabolism and RNA granules, however, are significantly emerging as novel potential targets for the comprehension of the molecular events leading to neuronal deficits. Indeed, defects in ribosome activity, ribosome stalling, and PQC—all ribosome-related processes required for proteostasis regulation—can contribute to triggering stress conditions and promoting the formation of stress granules (SGs) that could evolve in the formation of pathological granules, usually occurring during neurodegenerating effects. In this review, the interplay between proteostasis, mRNA metabolism, and SGs has been explored in a neurodegenerative context with a focus on Alzheimer’s disease (AD), although some defects in these same mechanisms can also be found in frontotemporal dementia (FTD) and amyotrophic lateral sclerosis (ALS), which are discussed here. Finally, we highlight the role of the receptor for activated C kinase 1 (RACK1) in these pathologies and note that, besides its well characterized function as a scaffold protein, it has an important role in translation and can associate to stress granules (SGs) determining cell fate in response to diverse stress stimuli.

## 1. Introduction

Over the last century, an increased incidence of neurodegenerative diseases including amyloidosis, tauopathies, α-synucleinopathies, and transactivation response DNA binding protein 43 (TDP-43) proteinopathies has been observed along with an increase in the average lifespan [1]. Neurodegenerative disorders are a heterogeneous group of pathologies characterized by a progressive structural and functional degeneration of the central and/or peripheral nervous systems. These diseases are classified considering their clinical presentations, such as extrapyramidal and pyramidal movement disorders and cognitive or behavioral disorders. From a biochemical point of view, neurodegenerative diseases are typically characterized by protein accumulations that lead to a progressive neuronal dysfunction. As a consequence of proteotoxic stress, alterations in ubiquitin–proteasome and autophagosome/lysosome systems, oxidative stress, and neuroinflammation in these protein aggregates contribute to neuronal death [1]. One of the markers of neurodegenerative diseases is represented by pathogenic protein inclusions with hydrophobic and aggregation-prone features termed amyloid proteins (APs). These APs can be specific to one disease, such as amyloid β (Aβ) in Alzheimer’s disease (AD), or found in various neurodegenerative conditions, such as α-synuclein or phosphorylated TDP-43. The APs are only partly cleared by autophagy and the ubiquitin–proteasome system (UPS). Despite their different structures, APs are hypothesized to be generated by a common pathological pathway of the misfolding process [2]. Proteins undergo misfolding from their native states to form intermolecular β-sheet-rich structures, ranging from small oligomers to large fibrillar aggregates that accumulate in the diseased brain [3]. Neurons are particularly vulnerable to the toxic effects of mutant or misfolded proteins. Several pieces of evidence suggested that protein misfolding, followed by oligomerization and accumulation of APs in the brain, are the main triggers of pathological alterations responsible for the development and progression of different neurodegenerative diseases. Aggregation of misfolding-prone proteins is thought to have a preeminent role in neurodegenerative diseases research [4]. In addition, the well-studied role of extracellular and intracellular proteins, the important role of RNA metabolism, and the formation of RNA granules have also recently emerged in neurodegeneration research [4].

The RNA granules are membrane-less organelles generated through liquid–liquid phase separation (LLPS), a process consisting in the formation of liquid water droplets in response to a high concentration of molecules interacting through weak intermolecular hydrogen bonds. The RNA granules include cytoplasmatic granules, namely stress granules (SGs), processing bodies (P bodies), transport granules, storage granules, activity-dependent granules, and myo-granules, and also nuclear bodies (nucleoli, Cajal bodies, nuclear speckles, and paraspeckles). In neurodegenerative diseases and myopathies, SGs are the most relevant RNA granules, although they have been also found to be implicated in cancer, inflammatory disorders, and viral infections [4]. For a long time, SGs have been considered as an adaptive response to a transient stress. However, it has been demonstrated that chronic illness generates a persistent stress that leads to the maturation of SGs— i.e., the multi-step process required for translation factors, mRNAs, RNA-binding proteins (RBPs) and other proteins to coalesce into a primary nucleated SG (reviewed in [4])—in more stable complexes that might lead to the formation of pathological SGs, usually occurring during neurodegenerating events [4]. Defects in ribosome-related processes required for proteostasis regulation, such as ribosome activity, ribosome stalling, and protein quality control (PQC), can contribute to triggering stress condition, promoting SG formation that could evolve in the formation of pathological granules.

The aim of this review is to discuss the interconnection between different aspects of proteostasis deregulation and SG formation in response to the stress conditions that occur in neurodegenerative diseases. The focus of this work is principally on AD, although some defects in these same mechanisms can also be found in frontotemporal dementia (FTD) and amyotrophic lateral sclerosis (ALS), which are also discussed. Finally, we highlight the role of the scaffold, ribosomal, translation- and SG-correlated protein receptor for activated C kinase 1 (RACK1) in these pathologies.

## 2. Translation Impairment in Neurodegeneration

Protein synthesis is a strictly controlled molecular process because of its central role in different key cellular events, including homeostasis maintenance and response to extra- and intracellular cues. Increasing evidence suggests a dysfunction of the translation machinery in different neurodegenerative disorders. These dysfunctions are characterized by the accumulation of pathological protein aggregates, which could reflect defects in both ribosome and ribosome-associated activities.

### 2.1. Ribosome Dysfunction and Impaired Protein Synthesis

Several studies have reported that alterations or defects of protein synthesis may occur in AD [5,6]. In this regard, direct evidence from both mild cognitive impairment (MCI) and AD patients, particularly in brain areas involved in cognition, revealed a ribosomal dysfunction characterized by decreased protein synthesis and RNA alterations [7], while initiation factors levels were not altered in these same brain regions [8].

Emerging data suggest that translation elongation plays a role in AD onset and defects in protein synthesis compromise neuronal functions, favoring AD development by affecting the correct translation mechanism [9,10]. The microtubule-associated protein tau abundantly associates with ribosomes in human brains of AD patients compared to healthy brains, leading to a decreased translation. This aberrant association also impairs the synthesis of pivotal synaptic proteins, contributing to synaptic dysfunction. These pathological associations between tau and ribosomes in the AD brain results in a reduction in nascent proteins, including those required for synaptic plasticity, central for memory and learning. Therefore, this observation links the appearance of pathologic tau inclusions with cognitive impairments featured by all tauopathies, including AD and FTD [11]. In mouse models of FTD-tau—tauopathy caused by aberrant changes of tau—a mass spectrometric analysis revealed mutant tau-induced ribosome alterations and a decrease in specific ribosomal proteins (RPs), leading to a reduction in protein synthesis and ribosome biogenesis [12]. Altogether, these observations suggest that impaired ribosome functions may arise even after correct ribosome assembly and maturation, hampering protein synthesis and increasing neuron vulnerability [13]. In addition, oxidized ribosomes were shown to directly induce a decrease in protein synthesis [11,14]. In this regard, increased ribosomal RNA (rRNA) oxidation has been observed in early AD [7,15] and high levels of oxidized rRNA were found in 40S and 60S ribosomal subunits of MCI patients and in mature 80S ribosomes of AD patients. These same analyses showed decreased levels of ribosome precursors in MCI patients and reduced levels of mature ribosomes in AD patients [15]. Most oxidation-related RNA damage occurs in brain areas prominently exposed to AD pathology and, in early AD, this is concomitant with the onset of cognitive decline [16]. Therefore, RNA oxidation also seems to take part in the dysregulation of the translation apparatus in this pathology [17]. Moreover, according to the MODOMICS database, other RNA modifications on coding and non-coding RNAs have been identified, although only a few have been linked to neurological disease [18,19]. These include pseudouridine, adenosine methylation at position 1 (m1A, also known as N1-methyladenosine), 5-methyl cytosine (m5C), and N6-methyladenosine (m6A). In this regard, m6A modifications have been shown to play a role in different processes including learning memory, neurogenesis, and axon regeneration. The dysregulation of m6A pathways has been implicated in the onset of neurological diseases including AD, where m6A modifications at the 3′-UTR of mRNAs alters the translation of transcripts linked to age-related disease phenotypes [20]. In accordance, recent evidence showed a progressive m6A increase concomitantly with AD severity in human brains. Mechanistic studies demonstrate that oligomeric tau (oTau) is connected to m6A-modified transcripts via heterogeneous nuclear ribonucleoprotein A2/B1 (HNRNPA2B1), which functions as a linker. Indeed, both m6A and m6A-oTau-HNRNPA2B1 complex levels are highly increased in brains of AD patients and in the P301S tau mice model, indicating that this complex favors the integrated stress response towards oTau [21]. The reversible m1A is known to target both rRNAs and tRNAs. This modification, which has been correlated to the increased tRNA structural stability and its correct folding, is decreased in brain tissues from an AD mouse model, where this reduced m1A methylation could impact translation efficiency. Therefore, the observed dysregulation of the m1A modification could contribute to AD aetiology by affecting protein synthesis [22]. Finally, a reduced polyribosome activity has also been linked to an increased RNA transfer (tRNA) oxidation—which alters their own stability and function—and changes in individual tRNA species, that in turn may also act as a compensatory mechanism to cope with intrinsic and extrinsic stressors, including oxidative stress [7]. In this regard, a mass spectrometry analysis on the cerebellum of AD patients identified a reduction in specific tRNA synthetases [23]. In addition to dysfunctional ribosomes and translation impairments as early events in AD pathogenesis [7], these finding suggest that decreased levels of tRNA synthetases may lead to a decreased global protein synthesis, hampering pivotal mechanisms required for learning and memory setting in AD.

### 2.2. Ribosome Stalling and Ribosome-Associated Quality Control

A pivotal step of mRNA translation is elongation, in which ribosomes scan the mRNA sequence to gradually form the nascent polypeptide chain. This process plays a crucial role in different aspects of protein synthesis, including differential expression, secretion, covalent modification, and co-translational folding [24,25]. Ribosome stalling, a local accumulation of ribosomes at specific mRNA codon positions, is involved in several physiologic processes, including mRNA degradation [26], modification of protein conformations [27], and regulation of protein expression [28], but also in pathological conditions [29].

**Figure 1:**
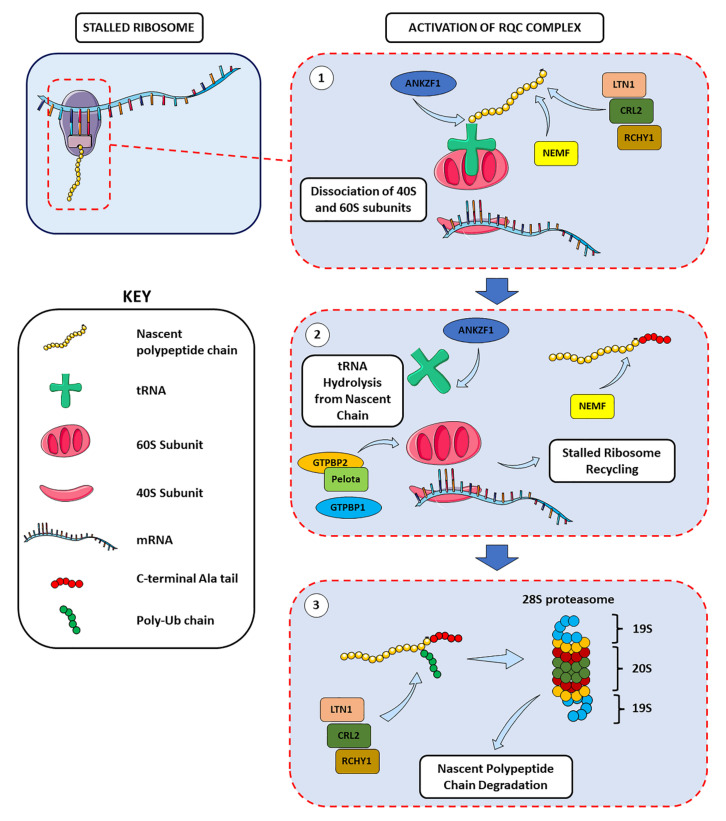
**The activation of the RQC machinery results in stalled ribosome resolution and degradation of aberrant nascent chains**. After scanning the arrested ribosomes, (1) the RQC complex induces the dissociation of the small and large ribosomal subunit, followed by (2) the hydrolysis of the tRNA from the nascent chain, the recycling of the translation machinery and, finally, (3) the degradation through the 28S proteasome of the aberrant nascent chains produced by stalled ribosomes. All these steps are achieved via a fine-tuned regulation of all the molecular players involved, and also thanks to the redundant action of specific proteins, which assures a complete and correct control over the possible errors of the translation process (see text for details).

Pivotal components of the ribosome surveillance machinery are collectively called ribosome-associated quality control (RQC). When translational errors induced by stalling take place, the nascent peptidyl-tRNA chains—which are retained by the 60S subunit—recruit the RQC machinery for stalled ribosome resolution. The RQC complexes scan the arrested protein synthesis machinery to recycle stalled ribosomes by inducing the dissociation of 40S and 60S ribosomal subunits, and degrade abnormal mRNAs and polypeptides [30]. Mechanistically, listerin E3 ubiquitin protein ligase 1 (LTN1) targets the polypeptide chain produced during ribosome stalling for its proteolytic cleavage, while the ankyrin repeat and zinc finger peptidyl tRNA hydrolase 1 (ANKZF1) hydrolyses the tRNA from the ubiquitylated nascent chain before its degradation [30,31,32] (Figure 1). Nuclear export mediator factor (NEMF) modifies the nascent polypeptide chains produced by nonstop mRNAs—major erroneous mRNAs in mammals—with a C-terminal tail mainly composed of alanine (CAT-tail) to assist their ubiquitination and promote their degradation [33]. In addition, the functionally redundant E3 ubiquitin ligases cullin-RING E3 ubiquitin ligase 2 with its adaptor KLHDC10 (collectively indicated as CRL2^KLHDC10^) and ring finger and CHY zinc finger domain containing 1 (RCHY1) target C-terminal degrons and are involved in a NEMF-mediated, LTN1-independent degradation of RQC substrates to signal proteolysis and resolve stalled ribosomes’ protein products [34] (Figure 1).

Failure of RQC is correlated with the persistence of unresolved stalled ribosomes and the sequestration of chaperone proteins, which interfere with the PQC system ultimately leading to and promoting protein aggregation [35]. This essential role of RQC in the proteostasis regulation has been linked with the proteotoxic effect of incomplete polypeptides produced by stalled ribosomes. Failure to degrade these aberrant nascent chains has been observed to be involved in mouse models of neurodegeneration [30,35,36]. Alterations in recycling stalled ribosomes in neurons have been linked to neurodegeneration, but the specific molecular mechanisms and signaling pathways triggered in response to ribosome stalling have yet to be completely elucidated. Recent evidence indicated that an inefficient RQC of ribosome stalling could be linked to the manifestations of AD hallmarks [37]. In AD mouse models and AD patients’ samples, during APP C-terminal fragment (APP.C99) co-translational translocation at the endoplasmic reticulum (ER) membrane, ribosomes stalled and activated the RQC machinery to resolve paused translation and ribosome collision. In case of inadequate RQC, aggregation-prone CAT-tailed APP.C99 induced autophagy and endolysosomal impairments, favoring the aggregation of Aβ peptides. These observations, together with the presence of RQC components at the Aβ plaque core, suggest a role of defective RQC of ribosome collision and stalled translation in AD pathogenesis [37]. Although RQC is a newly discovered mechanism, mutations in RQC components, such as LTN1 and NEMF have been shown to cause neurodegeneration [29,36] and a progressive development of motor neuron degeneration in ALS mice models [32]. In addition to impairment of central components of the elongation machinery, dysfunctional tRNAs have been observed to induce ribosome stalling, resulting in neurodegeneration. Alterations of tRNA levels due to genetic mutations can affect translation by impairing the elongation process. A single nucleotide mutation of n-Tr20—a brain-enriched arginine tRNA isoacceptor—in C57BL/6J mice models resulted in a severe impairment in tRNA processing and a reduction in its mature levels. These alterations led to a brain-specific increased ribosome occupancy at arginine AGA codons and abnormal ribosome stalling [29]. In addition, a n-Tr20 mutation associated with a mutation in GTP-binding protein 2 (GTPBP2)—a direct binding partner of pelota, a ribosome recycling protein—has been correlated with a significantly increased ribosome stalling at AGA codons, ataxia, and widespread neurodegeneration in the cerebellum, cortex, hippocampus, and retina areas [29]. Due to its homology to no-go/non-stop mRNA decay protein HSP70 subfamily B suppressor 1-like (HBS1L) and its interaction with pelota, GTPBP2 may play a pivotal role in rescuing and recycling stalled ribosomes. Here, GTPBP2 and pelota cooperate in in the resolution and recycling of paused ribosomes and the degradation of mRNA and nascent protein (Figure 1). While GTPBP2 can compensate n-Tr20 mutation-induced elongation defects, its absence exacerbates ribosome stalling, leading to neuronal death and neurodegeneration [38]. In addition, the GTPBP2 homologue GTPBP1 is involved in the same pathway. Its brain-specific loss during tRNA deficiency led to codon-specific ribosome pausing with consequent neurodegeneration [39]. Mutations in n-Tr20, GTPBP2, and GTPBP1, prior to neurodegeneration onset, were correlated with the following: (1) the activation of general control non-derepressible 2 kinase (GCN2, also known as eukaryotic translation initiation factor 2-alpha kinase 4, or EIF2AK4), resulting in increased eIF2α phosphorylation; (2) the upregulation of genes regulated by activating transcription factor 4 (ATF4), a pivotal transcription factor involved in the integrated stress response (ISR) pathway; (3) the decrease in mTORC1 signaling, ultimately leading to an increased stalled ribosome-correlated neuronal death [38,39]. This suggests the existence of a possible feedback loop between translation initiation, elongation defects, and ribosome stalling and of a pivotal crosstalk between RQC and PQC systems through the activation of surveillance pathways.

**Figure 2:**
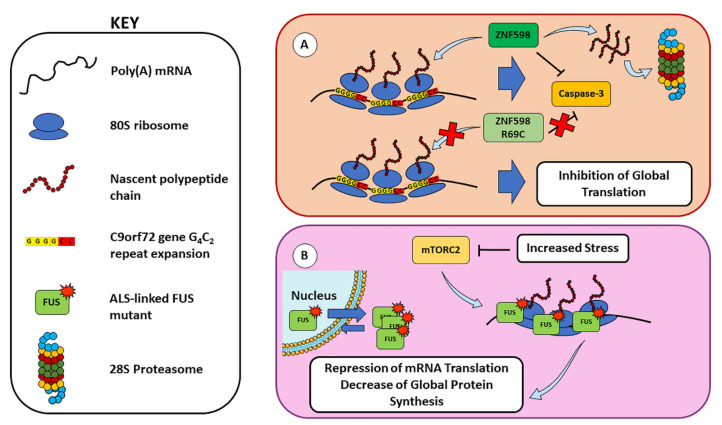
**RQC defects in FTD and ALS and their link with ribosome stalling.** (**A**) The E3 ubiquitin ligase ZNF598 co-translationally regulates the expression of the peptide chain derived from the G_4_C_2_ repeat expansion in *C9orf72* gene, by directing the aberrant peptides to the proteasome system and suppressing the caspase-3-mediated apoptotic pathway. However, the ZNF598R69C mutant observed in pathologic conditions showed a loss of these functions, resulting in translation inhibition. (**B**) In an ALS context, pathology-correlated mutants R521G and P525L of FUS have a higher cytoplasmic residency and can highly associate with translating ribosomes [42]. Upon mTORC2 inhibition, enhanced FUS association with polyribosomes results in the inhibition of global translation (see text for details).

Increasing evidence suggests a potential involvement of ribosome stalling also in the development of other neurodegenerative diseases, such as FTD and ALS. Indeed, elongating polyribosomes have been shown to stall on GGGGCC (G_4_C_2_) repeat expansion in the *C9orf72* gene, known to cause FTD and ALS (C9-ALS/FTD), leading to the production of neurodegeneration-driving dipeptide repeat proteins through repeat-associated non-AUG (RAN) translation and to translation inhibition [40]. In this regard, the RQC rate-limiting factor zinc finger protein 598, E3 ubiquitin ligase (ZNF598), has been shown to have a neuroprotective function in C9-ALS/FTD, since it co-translationally regulates the expression of *C9orf72*-derived protein to promote its degradation via the ubiquitin–proteasome pathway and to suppress proapoptotic caspase-3 activation, while ALS-linked mutant ZNF598R69C showed a loss of this function [41] (Figure 2A). In addition, the cytoplasmic residency of the RBP fused in sarcoma (FUS, also known as translocated in sarcoma, TLS) is prevalent in ALS and FTD and could contribute to the translational stalling of polyribosomes in an RNA-binding dependent manner [42]. Upon different stress conditions, mTORC2 signal transduction is compromised, leading to a reduced translation via FUS recruitment. The FUS negatively regulates translation through its association with polyribosomes and RNA in response to mTORC2 inhibition, and its cytoplasmic retention increases its proximity to polyribosomes for stalling to occur. This localization to stalled polyribosomes exerts a toxic repression of mRNA translation, resulting in a decrease in global protein synthesis [42] (Figure 2B).

Taken together, these data suggest that defects in recycling stalled ribosomes in the neuron may participate in the development of neurodegeneration, although further investigations are necessary to unravel the precise mechanisms by which ribosome stalling leads to neuronal death. In this regard, recent advancements in the analysis of ribosomal footprints in endogenous mRNA transcripts may prompt important improvements for a better understanding of elongation dynamics and for identifying endogenous sources of ribosome pausing and stalling.

### 2.3. Protein Quality Control and Proteostasis Regulation

The maintenance of a functional and stable proteome through a tight regulation of protein folding homeostasis is vital for cell survival, and the cell has different quality control strategies to monitor and maintain the proteome integrity. Following translation, newly synthesized nascent polypeptides are constantly at risk of misfolding and aggregation. The PQC is an essential cellular mechanism involving a network of molecular chaperones and protein degradation pathways that ensures protein homeostasis by degrading misfolded proteins and aggregates in a timely fashion. Newly folded proteins transit through the ER–Golgi apparatus for their eventual post-translational modification and secretion. Chaperones facilitate folding of proteins or refolding misfolded proteins, while incorrectly folded proteins are recognized by ER-associated degradation (ERAD), then targeted through different mechanisms, including the ubiquitin (Ub)-proteasome system (UPS) [43,44], the autophagy-lysosome system [45,46] and chaperone-mediated autophagy (CMA) [47].

**Figure 3:**
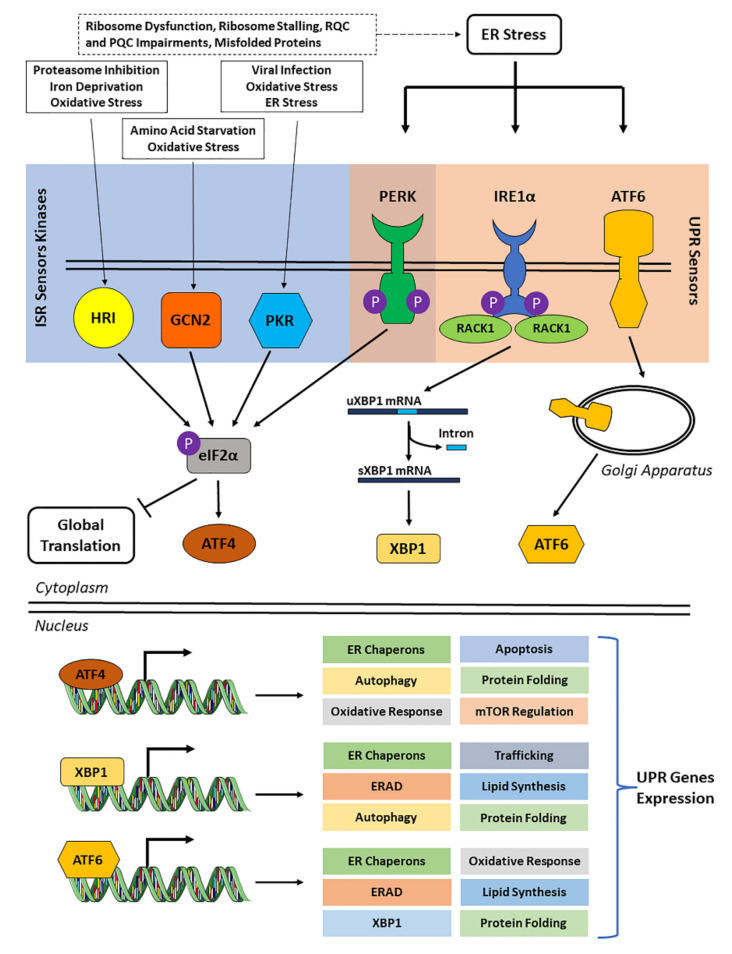
**The unfolded protein response (UPR) and the integrated stress response (ISR).** The accumulation of misfolded proteins in the ER leads to ER stress, which triggers intracellular stress sensors (UPR and/or ISR sensors) that can activate stress response pathways to maintain ER homeostasis. However, an excessive unresolved or chronic ER stress can trigger apoptosis responses leading to a different cell fate. The UPR sensors (PERK, IRE1α and ATF6) reside in the ER membrane, while ISR sensors (i.e., PKR, GCN2 and HRI) are cytoplasmic kinases that respond to different stressors than those of the UPR. The UPR and ISR pathways converge at the PERK sensor which, after its dimerization, halts global translation by phosphorylating eIF2α on Ser51. The IRE1α features a cytoplasmic kinase domain and RNase domain; upon dimerization and auto-phosphorylation, IRE1α induces its kinase and endoribonuclease activities, leading to unconventional splicing of X-box-binding protein-1 (XBP1) mRNA [55]. In this regard, RACK1 (receptor for activated C kinase 1) has been reported to activate IRE1α-XBP1 signaling pathway and induce neuroprotection in rat models [56], and the IRE1α-RACK1 axis has been shown to orchestrate cytoprotective responses after ER stress [57]. The ATF6 has a cytoplasmic domain which, upon ER stress, is processed in the Golgi apparatus, and an ATF6 fragment is released in the cytoplasm. After its activation, the UPR pathway induces central transcription factors ATF6, XBP1, and ATF4 that are redirected to the nucleus to mediate the expression of UPR downstream targets [55].

Due to its protein clearance activity, UPS plays a pivotal role in protein homeostasis during neurodegeneration by preventing protein misfolding and aggregation [8], and it regulates other several biological events, including transcription, DNA repair, cell cycle, and apoptosis [48]. In this context, molecular chaperones have a key role in proteostasis, as suggested by their protective role in the pathogenesis of neurodegenerative disorders in mouse models [49]. In this regard, they have been shown to inhibit the assembly of aggregation-prone proteins, such as Aβ and tau, and favor their UPS- or autophagy-mediated degradation [50]. In neurons, PQC and maintenance of proteostasis are demanding activities due to neuronal cellular structure and post-mitotic cellular state, which does not allow for the dilution of toxic substances through cell division. As a matter of fact, neurons are highly sensitive to misfolded proteins and their aggregates, and this susceptibility increases with their aging, as suggested by the correlation between PQC failure and neurodegenerative diseases [51,52]. Neurodegenerative pathologies, including AD, are characterized by misfolding and, consequently, abnormal aggregation of disease-causing or disease-developing proteins, such as Aβ and hyperphosphorylated tau. Production and accumulation of these protein aggregates—Aβ plaques in the extracellular milieu and tau neurofibrillary tangles in neurons—lead to an abnormal activation of cytoprotective mechanisms, including the unfolded protein response (UPR). The UPR is a complex mechanism associated with the ER and activated by three different molecular pathways involving inositol-requiring transmembrane kinase/endoribonuclease 1α (IRE1α), protein kinase R (PKR)-like endoplasmic reticulum kinase (PERK, also known as eukaryotic translation initiation factor 2-alpha kinase 3, EIF2AK3), and activating transcription factor 6 (ATF6). These signaling pathways lead to the transcriptional activation of UPR genes—including degradation proteins, redox enzymes, and several chaperones—and to eIF2α phosphorylation to suppress cap-dependent translation, ultimately resulting in SG formation. It is known that eIF2α can be phosphorylated by other kinases that collectively form the ISR, including PERK, PKR (also known as protein kinase RNA-activated, interferon-induced double stranded RNA-activated protein kinase or eukaryotic translation initiation factor 2-alpha kinase 2, EIF2AK2), GCN2, and heme-regulated eIF2α kinase (HRI, also known as eukaryotic translation initiation factor 1, EIF2AK1). The role of all four ISR kinases has been investigated in a neurodegeneration context, including AD. Their activation leads to a reduction in general translation and an increase in the expression of stress-related mRNAs, including β-secretase (BACE1) and ATF4. This accelerates the establishment of AD hallmarks, including tau phosphorylation, Aβ formation, and the induction of pro-apoptotic and autophagy pathways (reviewed in [53]). Furthermore, ISR kinases have been suggested to play important roles in AD development and progression for the following reasons: (1) PERK prolonged overactivation results in decreased protein synthesis, memory impairment, and neuronal loss, as well as in pathological tau phosphorylation and Aβ production [53]; (2) PKR is highly expressed and phosphorylated in AD brains, localized within and around neuritic and Aβ senile plaques and correlated with Aβ production and neurotoxicity-mediating neuroinflammation [54]; (3) GCN2 reduces global protein synthesis and upregulates stress-related mRNAs (e.g., ATF4 and BACE1), thus, accelerating Aβ production, tau phosphorylation, and neuronal apoptosis [53]; (4) HRI regulates BACE1 translation in glutamatergic hippocampal synapses, contributing to synaptogenesis and memory consolidation [53]. Therefore, these findings suggest an underlying dysregulation of the UPR and ISR mechanisms in neurodegenerative pathologies (Figure 3).

Indeed, although interrupting protein synthesis through these protective mechanisms can reduce cellular stress due to protein misfolding and aggregation, a persistent eIF2α-mediated arrest of global protein translation favors pathological SG formation, thus, negatively affecting neurons and interfering with the maintenance of their homeostasis and other neuronal functions. Defective ribosomal products (DRiPs) appear to be the most prominent species of misfolding proteins that accumulate inside SGs, and this correlate with the functionality of the PQC machinery and its players, which recognize damaged proteins and DRiPs and target them to the proteasome or autophagy, thus, maintaining the liquid-like properties of SGs, a process referred to as granulostasis, central for physiological SG behavior [58,59,60]. Impairments in PQC and SGs are major contributors in ALS/FTD pathogenesis, since mechanisms of protein folding and clearance are often dysfunctional in these pathologies [61]. Although mutations in different genes coding for proteins involved in UPS (that degrades soluble proteins with a short half-life) and autophagy (that degrades long-lived, misfolded, and aggregated proteins, as well as damaged organelles) [62] have been identified, mechanisms triggering aberrant SGs have also been shown not only in ALS/FTD, but also in AD [4]. One mechanism is represented by the alteration of cell signaling after mTORC1 sequestration, and defects in SG disassembly have deleterious consequences for cell viability by impairing protein synthesis and metabolic pathways, while sensitizing cells to apoptotic stimuli. When chaperone-mediated granulostasis fails to dissolve aberrant SGs, AN1-type zinc finger protein (ZFAND), the autophagy receptor SQSTM1/p62 and valosin-containing protein (VCP) can cooperate to degrade SGs via proteasome and autophagy, respectively. However, ALS/FTD mutations, such as in VCP, result in the accumulation of damaged proteins that, in turn, may indirectly favor their co-aggregation with SGs. Another mechanism through which SGs affect cell health is through sequestration of RBPs, that continuously shuttle between the nucleus and the cytoplasm to assist RNA transport and processing. Once in the cytoplasm, these RBPs show increased aggregation propensity, and this may enhance their sequestration inside SGs, promoting their conversion into an aberrant state. In ALS/FTD, TDP-43 and FUS mislocalization to the cytoplasm and their association to SGs represent a hallmark of these pathologies [63]. In addition, recent evidence reports that through a mechanism mediated by the RBP protein TIA1 and pathological SGs, oligomeric tau propagates toxic tau pathology, suggesting a broad role for SGs in the mechanisms of tau-mediated neurodegeneration [64,65]. Moreover, oTau pathology co-localizes with HNRNPA2B1 [21] and with other RPBs including PABP, HNRNPA0, eIF3η, and EWSR1 that are involved in tau-mediated neurodegeneration [4]. Indeed, during stress, an increased interaction between tau and mRNA is detectable in the somatodendritic arbour. The interaction of tau with SGs stimulates the formation of insoluble tau aggregates and has important consequences for the pathophysiology of tauopathies. These findings indicate that the physiology and pathophysiology of tau provide the biological link between RBPs and both SGs and the translational stress response [4].

Altogether, these considerations indicate that defects in UPS, autophagy, and vesicular transport contribute to ALS/FTD and AD pathogenesis, which is associated with the aggregation of misfolded proteins and the RBPs. In this regard, mutations and dysregulation of different RBP recruited into SGs can be either the primary cause or major contributors of neurodegenerative diseases [63]. Indeed, both genetic and experimental data reported a strong association between SG-recruited RBP protein aggregation and age-related pathologies.

## 3. Overview on Stress Granules: Function, Composition, Assembly, and Their Role in Neurodegeneration

Stress granules represent a conserved cellular strategy to minimize stress consequences and promote cell survival [66], since they are found in different organisms including yeast, plants, protozoa, Drosophila melanogaster, and mammalian cells [66,67]. The SGs are membrane-less transient structures that assemble in the cytoplasm in response to various stress stimuli, which include heat shock, nutrient deprivation, hypoxia, virus infection, oxidative stress, and UV radiation [68]. They are electron dense complexes that may display different diameter sizes, and their composition depends on cell type and source of stress.

Depending to the stress source, the following two types of stress can be identified: type 1, which is caused by stressors that are transiently applied, such as hypoxia, virus infection, arsenite, metabolic stress, oxidative stress, and heat shock, and type 2, which is caused by genotoxic drugs, nutrient starvation, and X-rays, namely stressors that last longer [69,70]. Differences in type and duration of the stress source decide the composition of SGs, depending on the inclusion or exclusion of different translational machinery components. In fact, while type 1 stressors can induce the formation of acute SGs, type 2 stressors promote chronic SGs aggregation. Both complexes are composed of the classic SG components, but chronic SGs lack several acute SGs-associated components, including RPS6 (ribosomal protein S6), 18S rRNA, and RACK1 [70,71].

The SGs are composed of miRNAs, polyA-RNA, 40S ribosomal subunits, translation initiation factors (eIF), RBPs, and signaling molecules [66,67,72,73,74,75,76]. In this regard, it has been reported that stress also causes translocation of many RBPs from nucleus to cytoplasm [66]. Under stress conditions, SGs quickly assemble and sequester proteins and non-essential transcripts, whose translation is transiently stopped [77], in order to exert a cytoprotective effect by shifting translation towards protective proteins and arresting global protein synthesis. This response is carried out to preserve energy, which is eventually used to promote shelter mechanisms, such as synthesis of DNA-repairing proteins and chaperones, against stress damage [78]. The cytoskeleton, and in particular microtubules, has a key role in both SG formation and clearance [79]. In fact, microtubule associated proteins (MAPs) are not only required for messenger ribonucleoprotein particles (mRNPs) transportation to SGs, but they also promote the fusion of smaller SGs into bigger ones [66,71].

The formation of SGs begins when global translation is shut down and initiation complexes separate from translating ribosomes. Meanwhile, granule nucleating proteins associate in the cytoplasm to form initial granules [80]. This aggregation is supported by proteins with prion-like domains (PLDs) and intrinsically disordered domains (IDDs), which are characterized by low-complexity sequences. These domains enable protein binding through electrostatic interactions and support liquid–liquid phase separations (LLPS), favoring SG assembly [66]. During SG formation, their size increases thanks to RBP aggregation, led by their low-complex sequences within PLDs and IDDs [81]. A variety of proteins, such as G3BP1 (Ras GTPase-activating protein-binding protein 1), TIA1, fragile X mental retardation protein (FMRP), TIA1-related protein (TIAR1), and tristetraprolin (TTP), share several properties necessary for primary SG aggregation. These proteins can initiate SGs assembly through binding RNA and untranslated mRNA [66,81,82], and they recruit other proteins through PDLs and IDDs, which serve as scaffold platforms to establish the interactions. Secondary aggregation, which involves SG nucleators, is exerted by a variety of hetero-oligomeric complexes that promote SG formation and growth [78]. This mechanism is stimulated by PABP1, which binds and connects different SG components, which include stalled translation initiation complexes and SG core proteins. In the final step of SG production, core SG proteins recruit other factors, such as ATP-dependent protein remodeling complexes [78] and signaling molecules [66,79], leading to the formation of mature SGs.

The formation of SGs can be promoted either by an eIF2α-dependent or an eIF2α-independent pathway. In the eIF2α-dependent pathway, Ser51 phosphorylation on eIF2α prevents GDP/GTP exchange in the GTP-eIF2α-tRNAMet complex, which mediates the binding between initiator tRNA, the 40S ribosome subunit, and eIF2α. This phosphorylation, which is mediated by different kinases, notably PKR, HRI, PERK, and GCN2, sees each kinase involved in response to a different stressor, and prevents the generation of the 43S pre-initiation complex and inhibits cap-dependent translation. In the eIF2α-independent pathway, SGs are formed due to mTOR inhibition or interference with eIF4F. This complex, formed by eIF4A, eIF4E, and eIF4G, together with eIF2-α, represents a crucial point for the control of translation initiation [4].

Given the transient nature of SGs, they disassemble and global translation is restored when the stress source is removed [4]. The disassembly of SGs is mediated by two different mechanisms, namely autophagy and chaperone-mediated disaggregation [81]. Granulophagy, the autophagy-mediated mechanism that removes SGs [83], relies on VCP, an ATPase required for the disaggregation of SG proteins. In this process, vesicles incorporate SGs, which are eventually removed. As for the chaperone-mediated disaggregation mechanism, most studies indicate the HSP70 family as the main molecular players involved in the process [66,79,84,85]. Here, SGs play a role in controlling and deciding cell fate, since they can promote different pathways involved in regulating translation and cell survival [66,71]. In fact, they can interact with a variety of proteins, each leading to a different response.

Defects in SG accumulation and molecular composition have been observed in a variety of diseases, including neurodegenerative disorders [4]. These include AD, ALS, and FTD [86,87]. Mutations in SG proteins FUS, EWS, TDP-43, and tau, and the absence of granulophagy, appear to correlate with these neurodegenerative disorders [59,61,66,68,86,87].

Altogether, the considerations presented so far strongly indicate that an aberrant proteostasis regulation due to a dysfunction of ribosomes, UPR, RQC, or PQC can lead to the formation of SGs in neurodegeneration [4]. In this context, the receptor for activated C kinase 1 (RACK1) emerges as a possible bridge between translational impairment and SGs, thanks to its ribosome-related functions and its role in the neurodegenerative diseases here considered.

## 4. Receptor for Activated C Kinase 1

Among all ribosomal proteins found up- or down-regulated in different pathologic conditions, including AD [13], RACK1 emerges as an interesting player due to its pivotal roles in both physiological and pathological conditions and in different cellular settings, including the immune system [88,89,90,91,92], several cancer types [93,94,95,96,97,98], and in the neuronal context [99,100,101,102,103,104]. Traditionally, RACK1 has been mainly considered as a scaffold protein, since it can interact with its binding partners in different locations within the cell, and it is also involved in transporting these proteins to other cellular districts. Indeed, RACK1 interacts constitutively or transiently with various proteins and complexes, and it is required for important cellular pathways, including proliferation, apoptosis, and transcription [105]. In addition to its characterized function of binding and stabilizing several activated protein kinase C (PKC) isoforms to enable their substrates’ phosphorylation [89,105], a ribosomal-associated RACK1 function is now emerging.

### 4.1. RACK1 Role in Translation and in Neuronal Biology

Besides its well-known and studied function as a scaffold protein and signaling hub, RACK1 has an important role in translation. In this context, RACK1 interacts with C-terminal Ser235 in eukaryotic initiation factor 6 (eIF6) [105], which is localized on the large 60S ribosomal subunit, and prevents translation initiation by sterically inhibiting 80S ribosome formation through its binding with the 60S subunit. Then, PKCβII-mediated Ser235 phosphorylation allows eIF6 and 60S subunit dissociation, promoting mature ribosome formation. It has been demonstrated that eIF6-PKCβII binding on 60S occurs as a result of their interaction to a nearby 40S subunit via RACK1 [106]. However, it is unclear whether this phosphorylation simultaneously involves the association between PKCβII and eIF6 on 40S-bound RACK1 [107,108,109]. Furthermore, RACK1 also plays a physiological role in coordinating the translational response upon the activation of the PKC–Raf–extracellular signal-regulated kinase 1/2 (ERK1/2) signaling pathway, providing a scaffolding function for PKCβII. In fact, the RACK1-PKCβII complex phosphorylates eIF4G1 at Ser1093 and eIF3a at Ser1364, leading to the stimulation of global protein synthesis [110,111]. The RACK1 binding to ribosome is fundamental for the full translation of capped mRNAs and eIF4E recruitment [112]. However, negative charge in the RACK1 loop increases swiveling of the 40S head domain, similar to several internal ribosome entry sites (IRESs), enabling human ribosomes to support eIF4A-independent translation [113]. The RACK1 function is also linked to the modulation of translation elongation and monitoring translation quality [110]. Indeed, RACK1 binding to the 40S subunit stabilizes both the activity and conformation of the 80S ribosome by monitoring the properties of nascent peptides, and the RACK1-40S complex might be required for RQC [110,114]. In this regard, the regulatory ubiquitylation of specific ribosomal proteins (Rps20, Rps10, and Rps3) required for ribosome stall resolution is catalyzed by ZNF598, which together with RACK1 is needed for the initiation of ribosome stall resolution in mammals [115].

At neuronal level, a decrease in RACK1 expression has been reported in the frontal cortex of AD patients, indicating a PKCβII anchoring deficit, which correlates with cognitive impairment and memory decline [99,100]. In the rat hippocampus, RACK1/PKCβII association has been reported to be involved in metabotropic glutamate receptor 1/5 (mGluR1/5)-triggered control of protein synthesis. It is also understood that PKCβII is activated via post-synaptic mGluR1/5 stimulation, increasing the levels of RACK1/PKCβII complexes bound to mRNP complex-associated polyA-mRNAs, modifying their phosphorylation pattern. Changes in mRNP’s phosphorylation have functional consequences for translation and may lead to the interruption of mRNP transport, demasking translational arrested mRNAs [116]. This results in changes in the translation efficiency of a subset of post-synaptic mRNAs, increasing mRNAs’ local concentration and leading to activity-dependent synthesis of specific proteins [117]. Altogether, these observations, in addition to RACK1 somatodendritic localization, indicate that synaptic activity may affect mRNP composition in dendrites and that post-synaptic mGLuR-coupled RACK1/PKCβII association allows for changes in translation efficiency spatially restricted to activated synapses, thus affecting only mRNAs close to them [117].

Altogether, the literature indicates that RACK1 activity may result in several effects on translation and in gene expression, since it can recruit proteins that regulate the translation of specific mRNAs through different molecular mechanisms [105]. In this regard, although RACK1 acts primarily as a ribosomal protein [105,118], it exerts also extra-ribosomal functions [94], playing a role in axon growth and guidance [119,120,121], local translation and point contacts in growth cones, and plays a potential role in neuroprotection [101,122].

### 4.2. Role of RACK1 in SGs

**Figure 4:**
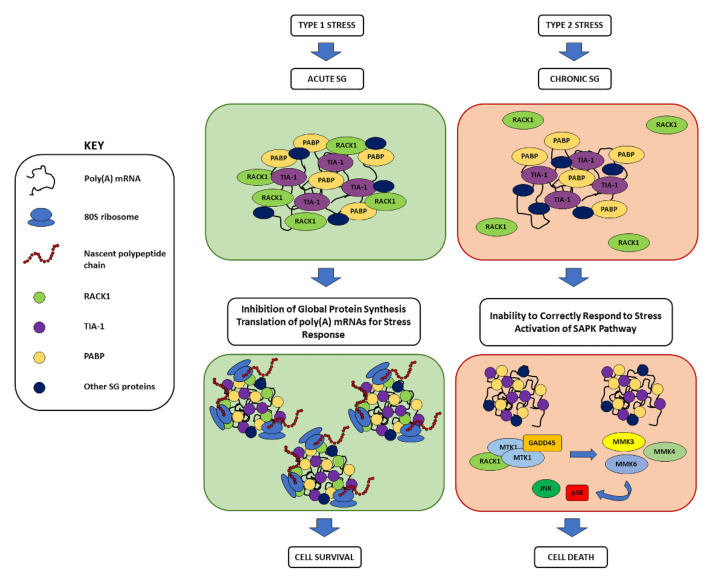
**Role of RACK1 in acute and chronic SGs.** Together with TIA1 (in violet), PABP (in yellow), and other SG-related RBPs (in dark blue), RACK1 (in green) has been recognised as a ribosomal protein recruited in SG formation. While included within acute SGs produced after type 1 stress promoting cell survival, RACK1 is excluded in chronic SGs after type 2 stress and remains in the cytoplasm, where it can activate the SAPK cascade, leading to JNK and p38 MAPK activation, which results in cell death (see text for details).

The presence of RACK1 in SGs changes their composition, which changes cellular fates; while acute, RACK1-containing SGs promote cell survival, and RACK1 is not sequestered in chronic SGs and remains, instead, in the cytoplasm, leading to the activation of stress-activated MAPK (SAPK) pathways and, ultimately, to apoptosis and cell death [69]. The SAPK cascade, triggered during chronic stress, involves several MAPK kinases, the growth arrest and DNA damage-inducible (GADD45) family of proteins, and RACK1. Among the kinases, MAP three kinase 1 (MTK1), MKK3, MKK6, MKK4, p38, and JNK have been observed. Instead, among the GADD45 family of proteins—whose expression increases under stress conditions and are implicated in growth arrest, DNA repair, cell survival, senescence, and apoptosis—GADD45α, GADD45β, and GADD45γ have been reported to take part in SAPK pathways [69]. After exposure to type 2 stressors, GADD45 proteins are expressed and bind to the GADD45-binding domain of MTK1, firstly causing the dissociation of the MTK1 C-terminal catalytic domain (KD) from the N-terminal auto-inhibitory domain (AID) and, secondly, exposing the coiled-coil dimerization domain (DD) in MTK1. After these structural changes, two partially activated KDs are brought together. Subsequently, MTK1 promotes the autophosphorylation of its DD, which results in a full activation of MTK1. Activated MTK1 phosphorylates and activates MKK3, MKK4, and MKK6, which in turn trigger p38 and JNK activation, leading to cell death [69,123]. The role of RACK1 in this pathway consists of binding MTK1 and facilitating its activation upon exposure to type 2 stressors. In the MTK1 structure, the RACK1 binding domain partially overlaps with the AID and GADD45-binding domain, suggesting that RACK1 may be specifically required for MTK1 regulation [124,125]. Indeed, RACK1 can bind two or more kinases together, promoting MTK1 dimerization and, in the absence of stress, RACK1 keeps dimerized MTK1 in an inactive state until GADD45 binding takes place, suggesting that the formation of the RACK1-MTK1 inactive complex enhances MTK1 activation by GADD45 [69] (Figure 4). This mechanism could be responsible of several pathological conditions, such as stroke, myocardial infarction, inflammation, cancer, and neurodegenerative disease, strongly suggesting that RACK1 plays a central role in the crosstalk between cell survival cascades and SAPK pathways [69,126,127].

### 4.3. SGs and RACK1 in Neurodegeneration

At the cellular level, neurodegeneration features neurons attempting to meet cell death, and it is often characterized by pathological inclusions, including Lewy’s bodies, Aβ plaques, NFTs, and SGs [128]. Although their formation supports the development of neurodegenerative disorders, it is still unclear if these inclusions are composed of SG proteins or if SGs proteins are themselves recruited to pre-formed inclusions. It has been proposed that pathological SGs are caused as a consequence of the misregulation of the SGs’ response or failure of SGs’ disassembly. In this context, it has been observed that neurodegeneration could be supported by the interaction between RACK1 and various pathological proteins within SGs [73,129,130].

In AD, RACK1 has been shown to have neuroprotective features [131] because of its role in promoting nonamyloidogenic processing by amyloid precursor protein (APP) via PKC activation [132]. This suggests the existence of a loop between the functions of APP metabolic products and PKC role, and that the dysregulated APP metabolism of several conditions, including AD, could have consequences on the potential protective functions of the non-amyloidogenic secreted APPα [99]. Indeed, RACK1 levels have been found to be decreased in post-mortem AD patients’ samples [99,101,133,134]—although discordant data were also reported [135]—suggesting a potential involvement of RACK1 in altered PKC activation associated with dementia. The depletion of RACK1 in the hippocampal neurons of mouse models has been shown to cause beclin-1 upregulation, resulting in induction of autophagy and impairment of learning and memory [136]. Moreover, an Aβ-induced loss of membrane-bound RACK1 in cortical neurons resulted in an impairment of muscarinic regulation of PKC and GABAergic transmission [137], which correlates with the previously observed Aβ-induced impairment in the regulation of GABAergic inhibition in prefrontal cortex—important for cognitive processes—regulated by muscarinic receptors through a PKC-dependent mechanism [138,139]. It is still uncertain if RACK1 plays a SG-related role in AD. Furthermore, the pro-inflammatory phenotype can lead to the formation of SGs [140,141,142,143] that have been found in AD patients, where their failed disassembly and persistence may play a role in the aetiology of this neurodegenerative disease [143].

Almost all ALS cases and over half of all cases of FTD are characterized by the cytoplasmic accumulation of SGs containing ubiquitin-positive and Ser409/Ser410 phosphorylated TDP-43 [4,144], an RBP involved in different processes that span from the regulation of RNA metabolism, transport, and translation of specific mRNAs [145,146,147,148,149,150,151,152] to SG formation [151]. The TDP-43 binds to the 40S subunit and increases eIF4E-binding protein 1 (4E-BP1) recruitment, a translational repressor protein that reduces the phosphorylation of eIF4E, preventing its binding to eIF4F and inhibiting translation [152]. In ALS/FTD patients, the development of TDP-43 inclusions appears to be caused by the failure of SG disassembly. Persistent SGs cause alteration of proteostasis, RNA homeostasis, and protein synthesis [153,154], finally leading to the deregulation of neuronal pathways. In response to stress, TDP-43 is translocated in cytosol and recruited within SGs. As stress persists, RNA-disassociated TDP-43 forms insoluble aggregates that tend to accumulate around SGs [4,144]. In ALS patients, RACK1 partially localizes within SGs, and TDP-43 acts a translational repressor for overall translation; its binding to polyribosomes via RACK1 could promote the formation of cytoplasmic inclusions under ALS-inducing conditions [152].

## 5. Conclusions

Alzheimer’s disease is the most prevalent neurodegeneration among the elderly. The main markers of this neurodegenerative disorder include amyloid plaques, neurofibrillary tangles, and dystrophic neurites. Based on these histological features and on a significant body of experimental evidence, most of the therapeutic approaches have been focused on counteracting protein misfolding and accumulation. New discoveries, however, are changing this perspective, leading the field to explore new altered pathways [155].

Impaired protein synthesis is a molecular event that occurs in neurodegeneration and that has been shown to have a link with the aggregation of unfolded proteins [156]. This deficit can in turn result in defective synapse transmission and organelle transportation [157]. Among the molecules that aggregate in complex insoluble inclusions, tau and Aβ have been the most studied. The physiological role of tau in regulating the structure and function of microtubules is clearly important for both the physiology and pathophysiology of neurons, but post-translational modifications of tau, including phosphorylation and caspase-dependent cleavage, are also critically important in the disease process [158,159]. Interestingly, it has been shown that ribosomes can associate to the tau protein and that, similarly to tau aggregation, the impairment of translation and ribosome dysfunction may represent one of first steps in AD progression [12]. Pharmacological studies in mouse models of prion and tauopathy disorders further reinforced this concept, as deficits observed in these models benefitted by pharmacological treatment aimed at restoring protein synthesis [160,161].

Beside the well-studied role of extracellular and intracellular proteins, the important role of RNA metabolism and the formation of RNA granules have also emerged in neurodegeneration. Together with the aforementioned link between tau aggregation and protein synthesis disruption, RNA oxidation has been reported to play a role in neurodegeneration. The analysis of oxidized RNA species detected in the brains of Alzheimer’s disease and amyotrophic lateral sclerosis patients revealed significant damage of mRNA and rRNA [162], which suggests a potentially beneficial antioxidant approach, both pharmacological and nutraceutical, to ameliorate these conditions [163,164,165,166].

As a consequence of such conditions, protein synthesis seemed to be substantially affected. Concurrent with protein synthesis and RNA metabolism alterations, a link between the assembly of RNA granules and ribosomal impairment has also been established in neurodegeneration [4]. Impairments in different mechanisms required for proteostasis regulation (e.g., RQC and PQC) have been associated with the neurodegenerative pathologies here considered, and it has been noted that these same dysfunctions could serve as triggers for SG formation. Therefore, RACK1 emerges as an interesting player not only because it is involved in the aforementioned mechanisms previously discussed and in translation, but also because it has been found as part of SGs. In this context, since ribosomal RACK1 levels are not altered in the brains of healthy aged mice [167], while its expression is decreased in AD [99,100], a pathological related reduction in RACK1 levels can contribute to the development of translation impairments. These, in turn, can lead to a chronic ER stress that, through the activation of UPR sensors, contributes to the enhanced eIF2α phosphorylation leading to pathological chronic SG formation.

**Figure 5:**
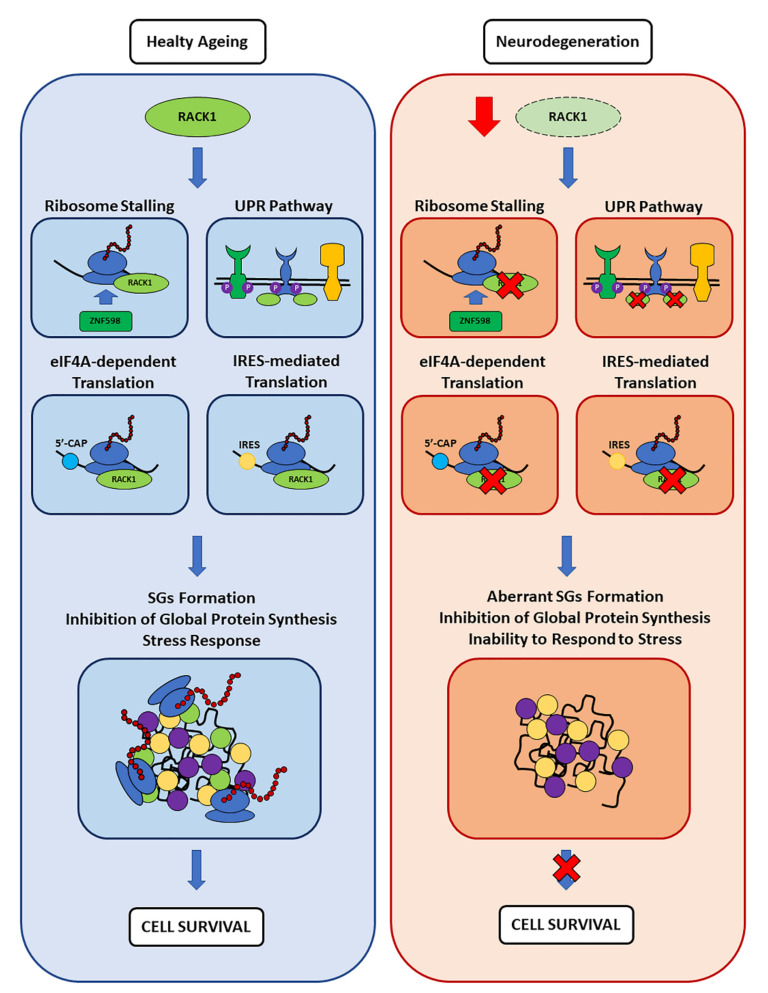
**Proposed model of RACK1 ribosome- and translation-related roles in healthy aging and neurodegeneration, and its impact on SG-correlated functions**. Here, RACK1 levels are reduced in AD patients compared to age-matched healthy controls [99,100] while its ribosome residency and stoichiometry are not altered during healthy aging [167]. Therefore, while RACK1 can contribute to the maintenance of proteostasis with its ribosomal and extra-ribosomal functions discussed here, its reduced levels in a pathological context can contribute to worsening the underlying proteostasis dysregulation observed in different neurodegenerative diseases.

The absence of RACK1 in chronic SGs together with its global pathology-associated reduction may comprise the activation of cell survival pathways (Figure 5). Deficits in RACK1 have been previously linked to memory impairment [99,100]. Therefore, we suggest that RACK1 might play a role in SG-regulated RNA metabolism/protein synthesis in neurons, and that such functions might play a part in many neurodegenerative diseases. In addition, we can speculate that RACK1 stabilization may be considered a potential therapeutic target in future studies aiming to reduce the progression of AD or other neurodegenerations.
